# Validation of the NPAQ-short – a brief questionnaire to monitor physical activity and compliance with the WHO recommendations

**DOI:** 10.1186/s12889-018-5538-y

**Published:** 2018-05-08

**Authors:** Ida Høgstedt Danquah, Christina Bjørk Petersen, Sofie Smedegaard Skov, Janne S. Tolstrup

**Affiliations:** National Institute of Public Health, University of Southern Denmark, Studiestræde 6, København K, 1455 Copenhagen, Denmark

**Keywords:** Population survey, Self-administrated, Open-ended questions, Closed-ended questions, Actiheart, NPAQ

## Abstract

**Background:**

Using self-reported surveys to monitor physical activity levels in the population require short items covering both time and intensity. The present study aims to 1) develop the Nordic Physical Activity Questionnaire-short from the original version of the NPAQ, 2) assess test-retest reliability and criterion validity of the NPAQ-short, and 3) test the NPAQ-short’s ability to monitor compliance with the WHO recommendations on physical activity. In addition, we aimed to compare open and closed-ended answering modes for the NPAQ-short.

**Methods:**

A sample of 122 participants were included. The NPAQ-short comprised of two questions on weekly moderate-to-vigorous physical activity (MVPA) and vigorous physical activity (VPA). It was filled in twice, two weeks apart, in open- and closed-ended versions. Physical activity was measured objectively by an Actiheart accelerometer worn 24 h/day seven consecutive days. Spearman’s rank correlation and Cohen’s kappa were used to assess correlations between the test and retest results, and between the objective and the self-reported measures.

**Results:**

Valid data was available for 92 participants. Test-retest reliability showed Spearman’s rho = 0.82 for MVPA and 0.80 for VPA. For the open-ended questions, the correlations between self-reported and objectively measured physical activity levels were Spearman’s rho = 0.33 for MVPA and rho = 0.32 for VPA. For closed-ended questions, the kappa-coefficients were 0.17 for MVPA and 0.21 for VPA. When using objective and self-reported measures to monitor WHO’s physical activity recommendations, the kappa correlations were 0.42 for open-ended and 0.34 for closed-ended answering modes.

**Conclusion:**

The NPAQ-short was found to be sufficiently reliable and valid to monitor physical activity levels in the population when using both open and closed-ended questions. However, using open-ended questions seems to be a better answering mode for self-reported surveys monitoring WHO’s physical activity recommendations.

**Electronic supplementary material:**

The online version of this article (10.1186/s12889-018-5538-y) contains supplementary material, which is available to authorized users.

## Background

Physical activity is a multidimensional construct measured by the duration, frequency and intensity of activities performed in different domains such as work or leisure [1]. Monitoring populations levels of physical activity in the general offers an opportunity to identify populations with physical activity levels below the official recommendations and track changes over time. The World Health Organization (WHO) recommends adults to be physically active for at least 150 min of moderate activity or 75 min of vigorous activity per week, or an equivalent combination of the two. [2]. Therefore, an instrument monitoring according to these guidelines needs to measure both duration and intensity. Often used self-reported survey instruments apply large questionnaire batteries (up to 27 questions) like the International Physical Activity Questionnaire (IPAQ) long version or the Global Physical Activity Questionnaire (GPAQ), which both have shown reasonable validity [3–6]. However, in large national surveys a brief or even single-item questionnaire is preferred as it reduces the complexity and therefore improves responding rates [7].

The Nordic Physical Activity Questionnaire (NPAQ) is a survey tool based on telephone interviews measuring physical activity by four questions [8]. Two of these questions consider duration of physical activity during the past week and are found suitable for monitoring the WHO recommendation on physical activity in populations in general. The two questions ask separately for duration of 1) moderate-to-vigorous and 2) vigorous physical activity combined during leisure time and transport. The remaining two questions considers number of days with moderate-to-vigorous and vigorous physical activity. They are thus not comparable with WHO’s recommendations on physical activity. For this reason, the NPAQ was adapted to a self-administered version that fit the purpose of a large national population based survey.

It is often discussed whether to use open-ended or closed-ended questions [7, 9, 10]. Closed-ended questions generally require less effort to answer; however, fixed categories offer less variation and may thus compromise further analysis and categories may affect how respondents perceive and answer the question [7, 9]. On the other hand open-ended questions pose a bigger task to the respondent which may ultimately result in non-response bias [7, 9]. Because of this ambiguity we wanted to compare the results of the two answering modes.

The main objectives of this study were to 1) adapt the interviewer-based questions from NPAQ into a two-question self-administered version, NPAQ-short, 2) assess test-retest reliability and criterion validity for NPAQ-short, and 3) test the ability of NPAQ-short to monitor compliance with the WHO recommendations on physical activity. Additionally, throughout the paper, we also aimed to compare open-ended and closed-ended answering modes for the two questions in the NPAQ-short.

## Method

### Study sample and recruitment

Sample size was estimated using the method developed by Walter et al. [11]. The aim of the study was to estimate correlation coefficients between self-reported and objectively measured physical activity between 0.3 and 0.6 [3, 4], which resulted in a sufficient sample size of 44 people [11]. To account for loss to follow-up and to be able to stratify by sex, the sample size was increased to 120 people.

Participants consisted of a convenience sample, recruited through different sources: an announcement was distributed by using the LinkedIn page of the National Institute of Public Health, by contacting specific workplaces and organisations, and through the extended network of the research group. The recruitment process was adjusted regularly, aiming for a balanced distribution in the study population regarding sex, age, education and region. All interested individuals received written and oral information about the project and signed a written informed consent. No incentives were given for participation, but participants received a report with their personal results afterwards.

The content of the survey did not require registration at the Regional Committee on Health Research Ethics, the Capital Region of Denmark (H-16020894). Data managing procedures was approved by the Danish Data Protection Agency (2015–57-0008). The survey content and procedures were designed according to the Helsinki Declaration [12].

### Physical activity questions

The interviewer-based questions in NPAQ were adapted into a two-question self-administered questionnaire, NPAQ-short. Both versions include one question on time spent on moderate and vigorous physical activity (MVPA) and one on time spent on vigorous physical activity (VPA). At first, questions from NPAQ were modified for self-administered use mainly by reducing the amount of text. This was done through discussions in the project group, existing literature and experience from other researchers.

**Fig. 1:**
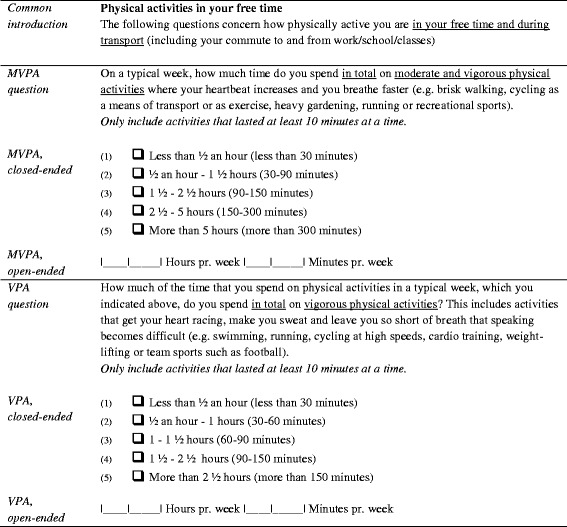
NPAQ-short in closed-ended and open-ended version. Translated from Danish

Then, content validity was assessed using cognitive interviewing, which gave insight into the cognitive processes taking place when answering the questions, in order to improve the wording of the questions [13]. A total of 12 cognitive interviews were conducted with participants of different sex, age, and education. The interviews considered e.g. the different elements of the questions (help text, descriptions of intensity, and examples of activities). From the interviews, it became clear that the respondents preferred different elements in their response process depending on their activity patterns, and thus all elements were included in the final version of the questionnaire. However, the cognitive interviews led to smaller adjustments in the wording of the questions. The final version of NPAQ-short is displayed in Fig. 1.

To assess test-retest reliability and criterion validity NPAQ-short was included in a questionnaire, which was distributed to participants twice, two weeks apart. Two versions of NPAQ-short were included in the questionnaire; first participants answered the closed-ended version and then, separated by additional questions, they answered the open-ended version. The additional questions concerned sex, age, weight, height, education, occupation, health status, sedentary behaviour, and self-rated fitness. The NPAQ-short with both open and closed-ended questions are displayed in Fig. 1.

At inclusion, participants answered the questionnaire on a computer or in some cases on a printed version due to technical reasons. After two weeks they received an e-mail with a link to the same questionnaire. A single participant without e-mail address received the second questionnaire by mail. Two reminders about answering the second questionnaire were sent out after one and two weeks.

### Activity monitor

Physical activity was measured using Actiheart (CamnTech Ltd., Cambridge, UK), which measures heartbeat and acceleration and combines the two into a measure of energy expenditure [14, 15]. Actiheart was placed horizontally on the chest using two electrodes (SP-50 ECG Electrode) and set to record in 60 s. epochs. Before long-term monitoring a signal test was conducted and participants completed a steptest, which was used for individual calibration. The steptest was administered from the Actiheart Software and lasted 8 min during which participants stepped up and down on a 20 cm step bench following a given beat with increasing intensity [16]. The participants received the Actiheart at the first visit and returned it after one week either picked by a member of the research team or returned by mail in a prepaid envelope. Participants wore the monitor 24 h/day for seven consecutive days however, they were asked to take off the monitor in case of skin irritation, swimming or any water-related sports (the monitor could be worn during showering). In addition, participants were instructed to change the electrodes every second day in order to ensure the best signal and to prevent skin irritation.

During the monitoring time period, participants kept a log in which they recorded sleeping patterns, working hours and when they changed electrodes. Irregularities, e.g. problems with the monitor or non-wear time were also documented.

### Data processing

Test-retest reliability and criterion validity were analysed for each of the two questions in NPAQ-short separately. This was done in order to assess the ability of the NPAQ-short to monitor physical activity duration and intensity. Additionally, answers to both questions were combined into a joint measure that was able to stratify the population according to the WHO recommendations for which criterion validity was analysed.

#### Questionnaire data processing

For open-ended questions hours and minutes of activity were summed. Inspired by the values used in the original version of NPAQ, weekly MVPA above 21 h and below 35 h were truncated to 21 h, and values above 35 h categorised as missing. Weekly VPA above 10 h and below 21 h were truncated to 10 h and values above 21 h were categorised as missing [17].

The WHO recommends to be physically active weekly for at least 150 min of MPA, or 75 min of VPA, or an equivalent combination (e.g. 100 min of MPA and 25 min of VPA). For objectively measured physical activity and open-ended questions compliance with WHO’s recommendations was calculated as a combination of MPA (MVPA-VPA) and VPA. Thus, participants were categorised as having physical activity levels above or below the WHO recommendations based on either MPA, VPA or a combination of the two. Thus, to have physical activity levels above the recommendations MPA/150 + VPA/75 should be equal to or above 1, while values below 1 classified as having activity levels below the WHO recommendations (Additional file 1). For the closed-ended version, participants were categorised as having physical activity levels above the standard recommendations if they answered MVPA categories 150–300 min and > 300 min, VPA categories 60–90 min, 90–150 min, > 150 min, or a combination of MVPA 90–150 min and VPA 30–60 min (Additional file 1).

In addition to the standard recommendation, WHO has an extended recommendation for additional health benefits. This recommendation consists of 300 min MPA, 150 min MVPA or an equivalent combination of the two. Calculation and classification for the extended recommendation is found in the Additional file 2.

Specificity and sensitivity show how well the open-ended and closed-ended questions identify the participants above or below the WHO physical activity recommendations. Specificity is the number of participants with physical activity levels below the WHO recommendations measured by both the self-reported and objectively as a percentage of those with levels below the recommendations according to the objective measure. Sensitivity is the number of participants with physical activity levels above the WHO recommendations both measured by self-report and objectively as a percentage of those with levels above the recommendations according to the objective measure.

### Activity monitor data processing

Actiheart files were trimmed in accordance with the logbook in the commercial software (Actiheart 4.0.116, CamNtech), using standard procedures for identifying non-wear time periods and interpolating gaps with missing data. Non-wear time was identified as a continuous period of > 2 h with no activity or with information from the logbook. Gaps < 5 min were filled by automatic interpolation based on previous and subsequent data [16]. The activity measure was calibrated using self-reported height and weight, resting heart rate as an average over the measured nights and results from the step test. Based on this, energy expenditure and MET-values for each minute of wear time was calculated using the branched equation model [18].

Combining this data with information from the log on sleep, work and leisure each minute was categorised into one of these three domains. Leisure time minutes with MET> 3.0 were summed into minutes of MVPA/week and leisure time minutes with MET> 6.0 were summed into VPA/week [2]. Only MVPA minutes in bouts of 10 or more minutes were included due to WHO’s recommendations on physical activity (allowing for 2 min below threshold) [19].

In line with previous studies, a workday had to have valid data for at least 4 h of work and 3 h of leisure time and a leisure day had to have at least 7 h of valid data (excluding sleep) to be included in the analyses [20]. Participants were included in the analyses if they had valid data for a minimum of three days, constituting of at least two workdays and one leisure day*,* or three leisure days in case participants did not work.

### Final study population

One hundred twenty two participants were included in the study, filled out the first questionnaire and had the Actiheart fitted. Of the 122 participants 115 filled out the second questionnaire. During data processing Actiheart data from 26 participants (21%) were excluded, e.g. due to technical problems with the device or lack of logbook data, 7 participants (6%) had less than the three required valid days. Participants with complete Actiheart data did not differ significantly from the total group of participants regarding age, sex, and education (data not shown). A few participants were lacking or had invalid questionnaire data which left 117 with valid data from questionnaire 1 and 108 from questionnaire 2.

This resulted in 105 participants with valid data for the reliability analysis, 92 with valid data for analysis of criterion validity of the MVPA question and 89 for the VPA question and compliance with the WHO recommendations.

### Statistical analysis

Spearman’s rank correlation coefficient (Spearman’s rho) was used to assess correlations between test-retest and between objective and self-report measures of MVPA and VPA. Based on results from similar studies Spearman’s rho ≥0.50 was considered acceptable reliability and Spearman’s rho ≥0.30 was considered acceptable validity [3, 4]. Validity was additionally considered using Bland-Altman plots.

For categorical variables correlations between objective and self-report measures of MVPA and VPA were assessed using Cohen’s Kappa. Taking existing literature into account, values ≥0.40 were considered acceptable reliability and values ≥0.20 were considered acceptable validity [21]. For variables with more than two categories a weighed Kappa was calculated. All statistical analysis were performed in Stata 14.1.

#### Sensitivity analysis

To test the robustness of the chosen cut-point for valid data, we made a number of sensitivity analyses repeating the main analyses. First, we used a requirement of 10 h/day of valid Actiheart data (instead of 7 h/day). Secondly, we conducted analyses with cut-point for valid data of 4 and 5 days respectively (instead of 3 days). In addition, because mean wear time was 5 days while the questionnaire covered 7 days, we repeated main analyses with standardised values of MVPA and VPA (standardised to a 7-day week with 5 workdays and 2 leisure days).

## Results

### Participants characteristics

Participants (50% men) had an average age of 43 years and were equally distributed on educational level (Table 1). Median time wearing the Actiheart was 5 days (Interquartile Range 4;5).

**Table 1:** Participants characteristics, *n* = 122

Questionnaire data	N (%)
Sex, men	61 (50)
Age, mean (min;max)	43 (17;85)
Educational level^a^	
Low	45 (37)
Medium	45 (37)
High	32 (26)
Labour market attachment	
Employed	70 (57)
Student	27 (22)
Unemployed/ retired	25 (21)
BMI > 25, kg/m^2^	45 (37)
Self-rated health; Less good/bad	7 (6)
Chronic disease (lasting > 6 mo)^b^	37 (30)
Functional limitations^c^	51 (42)
Inactive during leisure time	12 (10)
Physical activity, self-reported min/week (from questionnaire, *n* = 122)	
MVPA, closed-ended^d^	
≤ 150 min	55 (45)
≥ 150 min	67 (55)
VPA, closed-ended^e^	
≤ 60 min	61 (50)
≥ 60 min	61 (50)
MVPA, open-ended, median (IQR^f^)	210 (120;330)
VPA, open-ended, median (IQR^f^)	90 (30;120)
Physical activity, objectively measured min/week (from Actiheart, n = 92)	Median (IQR^f^)
MVPA	320 (160;549)
VPA	50 (11;114)

^a^Low: Basic school, vocational school or upper secondary. Medium: 3–4 years of higher education e.g. teacher, nurse, bachelor degree. High: > 4 years of higher education e.g. doctor, engineer, master’s degree

^b^Includes any illness, disease or disability lasting more than 6 months

^c^Experiencing any functional limitation during the past 6 months

^d^ ≤ 150 min: < 30, 30–90, 90–150 min. ≥150 min: 150–300, > 300 min

^e^ ≤ 60 min: < 30; 30–60 min. ≥60 min: 60–90; 90–150; > 150 min

^f^Interquartile Range (IQR):25;75

### Test-retest reliability

Correlations between the individual measures of physical activity (assessed by the questionnaires two weeks apart) were Spearman’s rho = 0.82 for MVPA and rho = 0.80 for VPA, for questions with the open-ended answering modes. Spearman’s rho for men were 0.88 for MVPA and 0.82 for VPA, and for women 0.78 for both MVPA and VPA (data not shown). For the questions with closed-ended answering modes, the kappa-values were 0.66 for MVPA and 0.59 for VPA (Table 2). For men, the Kappa correlation value was 0.57 for MVPA and 0.48 for VPA and for women the correlation was 0.73 for MVPA and 0.68 for VPA (data not shown).

**Table 2:** Test-retest reliability

	Spearman’s rho (ρ)	*P*
MVPA, open	0.82	< 0.001
VPA, open	0.80	< 0.001
	Cohen’s kappa (к^a^)	95% CI^b^
MVPA, closed	0.66	0.55;0.75
VPA, closed	0.59	0.47;0.69

Correlation between self-reported physical activity in questionnaire one and two (14 days apart). *n* = 105

^a^Weighted kappa

^b^Confidence Interval

### Criterion validity

Spearman’s rho was 0.33 for the correlation between self-reported and objectively measured MVPA and for VPA the Spearman’s rho was 0.32. For MVPA there were no differences between men and women however, the correlation for VPA was higher for men (Spearman’s rho = 0.43) than for women (Spearman’s rho = 0.23).

The Bland-Altman plots showed a mean difference between self-reported and objectively measured MVPA of − 111 min (limits of agreement, LOA: − 680;457) and 26 min (LOA:-173;-224) for VPA (Additional file 3). Distribution of the data appeared cone-shaped, illustrating that the higher the level of both MVPA and VPA, the larger the difference between the self-reported and objectively measured values.

For questions with the closed-ended answering modes, the kappa correlation value between the self-reported and the objectively measured MVPA was 0.17 and 0.21 for VPA. The correlations of questions with open-ended answering modes were kappa = 0.20 for MVPA and 0.20 for VPA (Table 3). In general the kappa values were higher for men (kappa = 0.25 for MVPA and 0.31 for VPA) than women (kappa = 0.12 for MVPA and 0.13 for VPA) (data not shown).

**Table 3:** Comparison between self-reported and objectively measured physical activity (min/week)

		*n*	Cohen’s kappa (к)^b^	95% CI
MVPA	Objectively measured vs. self-report closed-ended question	92	0.17	0.07;0.30
	Objectively measured vs. self-report open-ended question (categorized)^a^	92	0.20	0.07;0.36
VPA	Objectively measured vs. self-report closed-ended question	89	0.21	0.08;0.37
	Objectively measured vs. self-report open-ended question (categorized)^a^	89	0.20	0.06;0.34

Cohen’s Kappa (к) with 95% Confidence Interval (CI). Objectively measured activity has been categorized into closed-ended categories in order to calculate agreement

^a^Open-ended questions categorized into closed-ended categories to enable comparison

^b^Weighted Kappa

Kappa for agreement between the question using a closed-ended answering mode and a categorised version of the open-ended question of NPAQ-short was 0.50 for MVPA and 0.48 for VPA. In general participants were categorised with higher levels of physical activity when using the open-ended compared to the closed-ended versions of the questions (data not shown). This is also seen in Table 4, which shows that mean values of MVPA from the open-ended questions generally are above the maximum of the respective closed-ended category. The same applies to mean objectively measured activity. For VPA, mean values from questions using open-ended answering modes and objective measured physical activity are in general close to the limits of the respective closed-ended categories.

**Table 4:** Comparison of different categorised versions of self-reported and objectively measured physical activity

	Closed-ended	n	Open-ended, Mean (min/week) (SD)	Objective measures Mean (min/week) (SD)
MVPA (*n*=92)				
< 30 min		7	69 (64)	195 (264)
30–90 min		17	132 (104)	284 (194)
90–150 min		14	166 (75)	297 (235)
150–300 min		33	277 (108)	391 (233)
> 300 min		21	454 (322)	511 (246)
VPA (*n*=89)				
< 30 min		22	28 (41)	31 (44)
30–60 min		25	69 (44)	79 (76)
60–90 min		11	119 (27)	111 (112)
90–150 min		25	147 (65)	65 (66)
> 150 min		6	290 (155)	210 (97)

### Validity of compliance with WHO’s recommendations on physical activity

For objectively measured physical activity 78% of participants were categorised with a physical activity level above the WHO’s recommendations, 81% for open-ended questions and 73% for closed-ended questions. The correlation between the objective and the open-ended questions was kappa = 0.42 and for the closed-ended kappa = 0.34 (Table 5).

**Table 5:** Compliance with WHO’s recommendations on different physical activity measurement methods

		Self-reported		
Compliance with WHO’s recommendations	Objectively measured^a^ *n* (%)	Open-ended questions^a^ n (%)	Closed-ended questions^b^ *n* (%)	Open-ended questions categorized^c^ *n* (%)
No	20 (22)	17 (19)	24 (27)	13 (15)
Yes	72 (78)	72 (81)	65 (73)	76 (85)
Kappa (95% CI)	–	0.42 (0.19;0.65)	0.34 (0.12;0.56)	0.37 (0.14;0.61)
Specificity^d^	–	50%	55%	40%
Sensitivity^e^	–	90%	81%	93%

WHO’s recommendations on physical activity (> 150 min of MPA or > 75 min of VPA or an equivalent combination). Cohens Kappa (к) with 95% Confidence Interval (CI) for comparison between self-reported measures and the objectively measure. Specificity and sensitivity for self-reported measures compared to the objective measure. n = 89

^a^Calculated as a combination of minutes MVPA and VPA (described in Additional file 1)

^b^Combining both answers on MVPA and VPA-questions (described in Additional file 1)

^c^Open-ended questions categorized into closed-ended categories

^d^Specificity is the number of participants with PA-levels below the recommendations both measured by self-report and objectively as a percentage of those with PA-levels below according to the objective measure

^e^Sensitivity is the number of participants with PA-levels above the recommendations both measured by self-report and objectively as a percentage of those with PA-levels above according to the objective measure

The specificity, i.e. the proportion correctly classified as having physical activity levels below the recommendations, was 50% for open-ended questions, 55% for closed-ended questions. The sensitivity, i.e. the proportion correctly classified as having physical activity levels above the recommendations, was 90% for open-ended questions and 81% for closed-ended questions (Table 5). In general, analysis on the compliance with WHO’s extended recommendations showed lower kappa values (kappa = 0.33 for open-ended questions and 0.17 for closed-ended questions) (Additional file 4).

### Sensitivity analyses

Sensitivity analyses were carried out using different requirements for valid data: 10 h/day of valid data, 4 days of valid data and 5 days of valid data, and separately with standardised values of MVPA and VPA. None of the analyses changed the results markedly.

## Discussion

The present study tested the reliability and the validity of the NPAQ-short. The NPAQ-short was adapted from the NPAQ in order to monitor physical activity levels in populations as well as compliance with the current WHO recommendations on physical activity. The test-retest reliability of the NPAQ-short was above the predefined acceptable levels [3, 4, 21] (Spearman’s rho between 0.80 and 0.82, kappa coefficients between 0.59 and 0.66).

Regarding the validity of the self-reported physical activity measure compared to the objective physical activity measure, the questions with open-ended answering modes showed acceptable levels (Spearman’s rho 0.32 and 0.33 for MVPA and VPA respectively) [3, 4]. For the closed-ended version, only the question on VPA was found to be acceptable (kappa 0.21), while for the question on MVPA results were below the predefined level (kappa 0.17) [21]. Comparing open- and closed-ended answering modes, the kappa-values of 0.50 for MVPA and 0.48 for VPA indicated that the two versions correspond moderately [21]. In general, answers to the open-ended questions were closer to the objectively measured physical activity than answers to the closed-ended questions, especially for MVPA-values.

The Bland-Altman plots showed a mean difference for MVPA of − 111 min indicating that MVPA was underreported in the NPAQ-short compared to the results from the objective measure. Conversely for VPA, mean difference was 26 min indicating an overreporting of VPA. The cognitive interviews pointed out that some participants did not report activities like dog walking or walking to buy groceries as MVPA. This might explain why MVPA in general was underreported. On the other hand, the social desirability bias might lead to people overestimating their amount of physical activities in order to appear sporty and fit, which may explain why VPA was overreported.

The ability to monitor the compliance with WHO’s recommendations on physical activity showed high sensitivity (> 80%) and medium specificity (50 to 55%), implying that NPAQ-short was better at detecting people above the WHO recommendations than those below. In general, the open-ended version of the NPAQ-short tended to perform better than the closed-ended (kappa open-ended was 0.42 and closed-ended was 0.34).

Other studies testing the validity of questionnaire batteries like the IPAQ and the GPAQ have found correlations of Spearman’s rho between 0.27 and 0.49 [3–5]. The present study found similar coefficients (Spearman’s rho between 0.32 and 0.33). Other brief questionnaires assessing compliance with recommendations (30 min/day) found kappa values between 0.15 and 0.44 [19, 22], which are similar to the values in the present study (kappa between 0.34 and 0.42). MVPA was underreported by − 111 min (i.e. 15 min/day) this error is similar as found by others [23].

The NPAQ-short was modified and simplified from the interviewer-administered questionnaire NPAQ. The NPAQ has been validated with Pearson’s correlation coefficients *r* = 0.33 for MVPA and *r* = 0.23 for VPA [24], which are similar to the present study. When measuring compliance with the WHO physical activity recommendations, a study of the NPAQ [24] found higher levels of sensitivity (100%) than specificity (47%). These levels are similar to the levels found in the present study (81–86% and 50–55% respectively).

Overall, the NPAQ-short was found to have similar validity compared to other questionnaires however, our version benefits from being very brief which is an advantage in large population surveys. In addition, the NPAQ-short questionnaire is suitable for measuring the compliance with the WHO recommendations, which other brief questionnaires are not designed to.

Comparing the open-ended and closed-ended version of the questionnaire, little differences were observed in the ability to monitor physical activity. However, the open-ended question on MVPA tended to perform better than the closed-ended version. Regarding the compliance with the classifying the population according to WHO’s recommendations on physical activity, the open-ended questions were better at identifying participants *above* the recommendations, while the closed-ended were better at identifying participants *below* the recommendations. Olsson et al. have compared physical activity questions with open and closed answering modes and concluded that the closed-ended questions showed better correlations with objectively measured activity compared to the open-ended questions [10]. As described above, this was not the case in the present study. This can be due to differences in the phrasing of the questions and the answering categories. For instance the uppermost category in the questionnaire tested by Olsson et al. was > 2 h while the uppermost of NPAQ-short is > 5 h.

### Strengths and limitations

Strengths of the present study include its size, as the number of participants allowed for the analysis to be divided on sex. Additionally, the Actiheart monitor was used to measure physical activity objectively and thus it included a combination of accelerometry and heart rate. The general wear time was high (median = 5 days), and the Actiheart measure was calibrated using a steptest, thus the objective measure was regarded of high quality. Furthermore, we tested both open-ended and closed-ended questions on the same participants making comparisons between the two answering modes possible.

Limitations include the sampling method (convenience sample). Even though the population represented a variety of people regarding age, sex and education, questionnaire measures showed that the participants in general had better self-rated health, were better educated, and had a higher level of physical activity compared to the general population [25]. This might affect generalizability of the results as participants might be more motivated and more concerned about their physical activity level than the general population and thus better at self-reporting physical activity.

Finally, it is important to remember that the NPAQ-short was tested with the aim of monitoring physical activity levels in large national surveys and it might therefore not be suitable for individual feedback or intervention purposes.

When choosing physical activity questionnaires for surveys they should be robust for changes in recommendations on physical activity and they should be validated in similar populations. Therefore, it might be relevant to assess the validity of the NPAQ-short in an even larger populations outside the Nordic countries – and to reassess validity if the international recommendations on physical activity change.

## Conclusions

The original NPAQ questionnaire was adapted into a shorter and self-administered two-question version, NPAQ-short. NPAQ-short showed acceptable reliability and validity to monitor physical activity and compliance with WHO’s recommendations on physical activity in population based surveys. The open-ended version of the MVPA question showed higher agreement with objectively measured physical activity than the closed-ended version; however for VPA, open-ended and closed-ended answering modes were found to perform equally. Additionally, open-ended questions seemed better at measuring compliance to WHO’s recommendations.

### Perspective

When monitoring levels of physical activity in the general population it is often desirable to use the most low-cost, accurate, and robust questionnaires. This paper contributes with a short-item questionnaire (NPAQ-short). When comparing results on validity from validations studies of bigger and more time-consuming questionnaires such as NPAQ, IPAQ and GPAQ, this study of NPAQ-short has found similar or even better validity.

The NPAQ-short has already been applied to The National Danish Health Survey 2017 which gathers information about different health and morbidity measures within the grown-up population in Denmark. In the future, it would be interesting to assess the validity of NPAQ-short in other countries and settings.

## Additional files

Additional file 1: Calculation of participants’ compliance with WHO’s recommendations on physical activity. Calculation of participants’ compliance with WHO’s recommendations on physical activity (> 150 min of MPA or > 75 min of VPA or an equivalent combination) for continuously measured activity (from open-ended questions and objective measure) and answer categories from closed-ended questions. (DOCX 18 kb)

Additional file 2: Calculation of participants’ compliance with WHO’s extended recommendations on physical activity. Calculation of participants’ compliance with WHO’s extended recommendations on physical activity (standard: > 150 min of MPA or > 75 min of VPA or an equivalent combination, extended: > 300 min of MPA or > 150 min of VPA or an equivalent combination) for continuously measured activity (from open-ended questions and objective measure) and answer categories from closed-ended questions. (DOCX 14 kb)

Additional file 3: Bland-Altman plot of the difference between objective measured and self-reported physical activity and objectively measured physical activity. Bland-Altman plot of the difference between objective measured and self-reported physical activity on y-axis and objectively measured physical activity on x-axis, *n* = 92. Correlation between self-reported and objectively measured MVPA was Spearman’s rho = 0.33 (*p* = 0.001) and for VPA Spearman’s rho = 0.32 (*p* = 0.002). LOA = limits of agreement. (DOCX 24 kb)

Additional file 4: Compliance with WHO’s extended recommendations on physical activity. Compliance with WHO’s extended recommendations on physical activity (standard: > 150 min of MPA or > 75 min of VPA or an equivalent combination, extended: > 300 min of MPA or > 150 min of VPA or an equivalent combination) measured objectively and with open-ended and closed-ended self-reported questions. Cohens Kappa (к) with 95% Confidence Interval (CI) for comparison between self-reported measures and the objectively measure. Specificity and sensitivity for self-reported measures compared to the objective measure. *n* = 89. (DOCX 15 kb)

## Abbreviations

GPAQ: Global Physical Activity Questionnaire
IPAQ: International Physical Activity Questionnaire
MPA: Moderate Physical Activity
MVPA: Moderate and Vigorous Physical Activity
NPAQ: National Physical Activity Questionnaire
NPAQ-short: National Physical Activity Questionnaire - short
VPA: National Physical Activity Questionnaire - short
WHO: World Health Organization
