# Supplementary material for: Validation of the NPAQ-short – a brief questionnaire to monitor physical activity and compliance with the WHO recommendations
Source: BMC Public Health. 2018 May 8;18:601. doi: 10.1186/s12889-018-5538-y (PMC5941676; doi:10.1186/s12889-018-5538-y)
Supplement: Supplementary file 1 — Calculation of participants’ compliance with WHO’s recommendations on physical activity. Calculation of participants’ compliance with WHO’s recommendations on physical activity (> 150 min of MPA or > 75 min of VPA or an equivalent combination) for continuously measured activity (from open-ended questions and objective measure) and answer categories from closed-ended questions. (DOCX 18 kb) [file 12889_2018_5538_MOESM1_ESM.docx]

Additional file 1 Calculation of participants’ compliance with WHO’s recommendations on physical activity (>150 min of MPA or >75 min of VPA or an equivalent combination) for continuously measured activity (from open-ended questions and objective measure) and answer categories from closed-ended questions.

| Compliance with WHO’s recommendations | Calculation for continuously measured activity* | Answer categories from closed-ended questions |
| --- | --- | --- |
| No | (MPA/150 + VPA/75) < 1.0 | MVPA <30 min  MVPA 30-90 min  MVPA 90-150 min  VPA <30 min  VPA 30-60 min |
| Yes | (MPA/150 + VPA/75) ≥ 1.0 | MVPA 150-300 min  MVPA >300 min  VPA 60-90 min  VPA 90-150 min  VPA >150 min  MVPA 90-150 min + VPA 30-60 min |
| *MPA (Moderate Physical Activity) = MVPA-VPA | |  |
