# Supplementary material for: Validation of the NPAQ-short – a brief questionnaire to monitor physical activity and compliance with the WHO recommendations
Source: BMC Public Health. 2018 May 8;18:601. doi: 10.1186/s12889-018-5538-y (PMC5941676; doi:10.1186/s12889-018-5538-y)
Supplement: Supplementary file 3 — Bland-Altman plot of the difference between objective measured and self-reported physical activity and objectively measured physical activity. Bland-Altman plot of the difference between objective measured and self-reported physical activity on y-axis and objectively measured physical activity on x-axis, n = 92. Correlation between self-reported and objectively measured MVPA was Spearman’s rho = 0.33 (p = 0.001) and for VPA Spearman’s rho = 0.32 (p = 0.002). LOA = limits of agreement. (DOCX 24 kb) [file 12889_2018_5538_MOESM3_ESM.docx]

Additional file 3: Bland-Altman plot of the difference between objective measured and self-reported physical activity on y-axis and objectively measured physical activity on x-axis, n=92. Correlation between self-reported and objectively measured MVPA was Spearman’s rho=0.33 (p=0.001) and for VPA Spearman’s rho=0.32 (p=0.002). LOA = limits of agreement

|    |  |
| --- | --- |
