# Supplementary material for: Validation of the NPAQ-short – a brief questionnaire to monitor physical activity and compliance with the WHO recommendations
Source: BMC Public Health. 2018 May 8;18:601. doi: 10.1186/s12889-018-5538-y (PMC5941676; doi:10.1186/s12889-018-5538-y)
Supplement: Supplementary file 4 — Compliance with WHO’s extended recommendations on physical activity. Compliance with WHO’s extended recommendations on physical activity (standard: > 150 min of MPA or > 75 min of VPA or an equivalent combination, extended: > 300 min of MPA or > 150 min of VPA or an equivalent combination) measured objectively and with open-ended and closed-ended self-reported questions. Cohens Kappa (к) with 95% Confidence Interval (CI) for comparison between self-reported measures and the objectively measure. Specificity and sensitivity for self-reported measures compared to the objective measure. n = 89. (DOCX 15 kb) [file 12889_2018_5538_MOESM4_ESM.docx]

Additional file 4 Compliance with WHO's extended recommendations on physical activity (standard: >150 min of MPA or >75 min of VPA or an equivalent combination, extended: >300 min of MPA or >150 min of VPA or an equivalent combination) measured objectively and with open-ended and closed-ended self-reported questions. Cohens Kappa (к) with 95% Confidence Interval (CI) for comparison between self-reported measures and the objectively measure. Specificity and sensitivity for self-reported measures compared to the objective measure. n=89.

|  |  | Self-reported | | |
| --- | --- | --- | --- | --- |
| Compliance with WHO’s recommendations | Objectively measured* n (%) | Open-ended questions* n (%) | Closed-ended questions** n (%) | Open-ended questions categorized*** n (%) |
| No | 20 (22) | 17 (19) | 24 (27) | 13 (15) |
| Yes, standard | 13 (15) | 19 (21) | 29 (33) | 23 (26) |
| Yes, extended | 56 (63) | 53 (60) | 36 (40) | 53 (59) |
| Kappa (95 % CI) | - | 0.33 (0.16;0.51) | 0.17 (0.04;0.33) | 0.31 (0.16;0.48) |
| *Calculated as a combination of minutes MPA and VPA (described in eTable2)  **Combining both answers on MVPA and VPA-questions (described in eTable 2)  ***Open-ended questions categorized into closed-ended categories | | | | |
